# Selection and Exploration of Cultural and Creative Tourist Attractions Based on BP Network

**DOI:** 10.1155/2022/4386357

**Published:** 2022-01-29

**Authors:** Nian Xing

**Affiliations:** School of Journalism and Communication, Sichuan International Studies University, Chongqing 400031, China

## Abstract

In order to improve the selection and exploration effect of cultural and creative tourist attractions, this paper applies BP network to the selection and search of cultural and creative tourist attractions. Moreover, this paper improves the algorithm to promote the interrelation of cultural and creative industries and tourism industries to promote each other to form a complex and changeable dynamic system and proposes a personalized recommendation model for tourist attractions based on domain adaptation. Domain adaptation can effectively reduce the distribution difference between different data. In addition, this paper combines experimental research to verify the system model of this paper. The research results show that the cultural and creative tourist attractions selection search system based on the BP network proposed in this paper has a good selection effect of tourist attractions and has an important role in promoting the development of cultural and creative tourism.

## 1. Introduction

At present, creative products based on regional culture are mostly concentrated in art exhibitions and high-end crafts, which make the audience group smaller. Tourism products on the traditional market have low cultural content and poor product quality, and a large part of the products are direct applications of pictures, so there is no innovation. Domestic and foreign scholars are doing more and more research studies on regional culture, but the design and application of regional cultural elements are in the initial stage. Therefore, it is necessary to start with cultural elements and develop and design tourism cultural and creative products based on regional culture as the research content. Moreover, it is necessary to analyze the cultural elements with the most regional characteristics through the study of regional culture, conduct research and analysis on them, and establish a material library of regional cultural elements, which is conducive to increasing the added value of products and is conducive to product serialization and branding.

At present, many tourism, cultural, and creative products centered on regional culture are seriously homogenized, and many products in scenic spots only replace the parts of the products that can be pasted, resulting in similar products with little difference. Most tourism products have no sense of design, messy types, and no special features. Except for the different pictures, the product modeling functions of the direct map products are almost the same. On the surface, it is because tourism products lack cultural connotation. At a deeper level, it is that the level of cultural excavation is not enough. New technologies and new media are used to rearrange and utilize the regional culture, build a regional cultural material library, and preserve the culture at the same time. More people know the regional culture. Second, the establishment of a regional cultural material library is conducive to the development of the cultural industry, thereby supporting the development and iteration of subsequent cultural creative products. Third, through the study of regional culture, establish a relevant cultural research system, explore relevant research methods and research models, and apply them to the design and research and development of cultural creative products to guide the development of cultural creative products, upgrades, and changes in the current market, so that the products are no longer low quality and low culture. Because of its particularity, regional culture also represents ancient Chinese culture at a certain level. Using regional cultural elements to develop cultural and creative products will help promote the public to understand regional culture and history and understand Chinese culture. It can not only promote the development of local culture but also enhance tourists' awareness of local culture, thereby driving regional economic development. Development is more conducive to the establishment of national cultural self-confidence.

Regional culture refers to the cultural connotation with regional characteristics within a region containing material and spiritual characteristics, including the development form and social lifestyle of the place from time to time, as well as the unique cultural heritage and material heritage [1]. Natural resources and human resources are integrated into a cultural characteristic that can reflect national characteristics, thoughts, concepts, customs, beliefs, morals, ethics, and so on. Hundred schools of thought, piano, chess, calligraphy and painting, literature, drama, festivals, folk houses, architecture, characters, language, costumes, stories, characters, customs, dialects, singing and dancing, food, traditional Chinese medicine, religion, belief, philosophy, craftsmanship, martial arts, and other diverse regional cultures constitute the broad Chinese culture [2].

Packaging design research based on regional culture is to study the application of regional culture in packaging design. Taking Baminjin series packaging design examples as the starting point, it explores research approaches suitable for Baminjin packaging design, and through the inheritance and development of Bamin regional culture, it reflects the value of cultural innovation and wins a better investment market. It is necessary to apply regional culture in product packaging design and design according to consumers' cultural identity and emotional bonds of the brand, so as to enhance the cultural value of packaging design [3].

This paper applies the BP network to the selection of cultural and creative tourist attractions and builds an intelligent system based on the actual situation to promote the further development of cultural and creative tourism.

## 2. Related Work

For the study of cultural and creative industries, there have been relatively mature and systematic research results abroad. Literature [4] awakened the definition of the concept of creative industries based on the perspective of cultural economics. Literature [5] analyzed and compared the similarities and differences between the two terms “cultural entrepreneurship industry” and “creative industry” and pointed out that they have essentially the same characteristics. Literature [6] expanded the extension of the creative industry and collectively referred to the cultural industry and the art industry as the creative industry. Literature [7] analyzed the driving factors of innovation and pointed out that market demand plays a key role in guiding innovation, but it also restricts the development of innovation. Compared with technology, market demand has a greater driving role. Literature [8] put forward the “dual-factor theory” for the development of cultural and creative industries and believed that supply and demand are the two main driving forces that promote the development of entrepreneurial industries. Literature [9] systematically proposed the theory of creative economics, which led to the vigorous development of theories of creative economics. Literature [10] studied the industrial characteristics of traditional handicrafts and pointed out that cultural creativity can give handicrafts more added value. Literature [11] combined creative industries with tourism and pointed out creative tourism, a new idea for the development of tourism. Literature [12] believed that under different economic environments, entrepreneurial industries should apply different development models and advocate different models. The former focuses on market orientation, while the latter believes that government leadership can better promote the development of creative industries. Literature [13] defined industrial agglomeration, took major cities in the world as the research object, and defined the new urban development model of “creative cities.” Literature [14] compared several major cultural and creative industry development models. Literature [15] pointed out the evolution trend of Singapore's cultural and creative industries based on the perspective of potential mining. Literature [16] comparatively analyzed the creative industry, cultural industry, and cultural creative industry and sorted out the main similarities and differences of the three. Literature [17] analyzed the main influencing factors driving the development of cultural and creative industries based on the perspective of industrial competitiveness and further analyzed the value of cultural and creative industry chain research. Literature [18] studied the value chain creation mechanism of cultural and creative industries. Literature [19] pointed out that human resources, market demand, institutional environment, and basic environment are the key factors that promote the development of cultural and creative industries. On this basis, a framework of influencing factors of the cultural entrepreneurship industry is constructed. Literature [20] pointed out that the policy environment and cultural environment play a key role in the development of Beijing's cultural and creative industries.

## 3. Recommendation Algorithm Based on BP Neural Network Model

According to the above conclusions, it is known that the deep convolutional neural network cannot process data in non-Euclidean space and loses its effectiveness when encountering topological structure graph data. However, graph structures are common in real life, so more and more people are beginning to explore how to define convolution operations for graph data.

The main challenges of using convolutional neural networks for graph structures are as follows:

1. The graph structure data do not satisfy the translation invariance; that is, the structure of each node is different, and the number of connected neighbor nodes is uncertain, so it is impossible to use a convolution kernel of the same size to perform convolution operations.
2. The graph structure data have directionality. For example, users in social networks are connected.

The graph convolutional neural network for non-Euclidean spatial data is divided into two directions: spectral domain method and vertex domain method.

The vertex domain method of graph convolutional neural network has two ideas. One idea is to design convolution kernel and convolution operation based on graph structure data, and the other idea is to convert graph structure data into Euclidean space data structure.

Like the traditional convolutional neural network, the goal of the BP neural network model is to learn a function to extract features. Each neural network layer can be written as a nonlinear function:

(1) $$\begin{array}{c} H^{\left(l+1\right)}=f\left(H^{l},A\right),H^{\left(0\right)}=X,H^{\left(L\right)}=Z. \end{array}$$

Among them, *L* represents the number of layers, and *f*(·; ) of each layer differs only in parameters, and *Z* represents the final output.

(2) $$\begin{array}{c} f\left(H^{\left(l\right)},A\right)=\sigma \left(AH^{\left(l\right)}W^{\left(l\right)}\right). \end{array}$$

**Figure 1:**
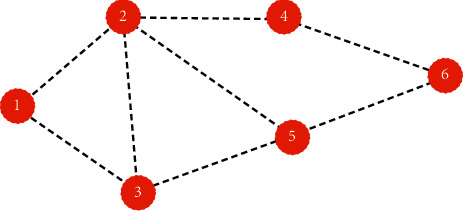
Graph structure data.

The feature matrix, adjacency matrix, and weight matrix are subjected to matrix multiplication to reflect the physical meaning of convolution. Obviously the weight matrix is easy to understand. Multiplying the adjacency matrix and the feature matrix is to learn the traditional convolution operation. The traditional convolution operation is to sum the product of the convolution kernel and the corresponding element. The convolution operation of the graph structure also needs to meet the translation invariance; that is, the number of elements in the convolution kernel participating in the convolution operation must change according to the graph structure, and the translation invariance in the graph structure is reflected in the adjacency matrix. Graph structure data are as shown in Figure 1.

(3) $$\begin{array}{c} X=\left[\begin{array}{c} a_{11},a_{12},a_{13},\ldots ,a_{16}\\ a_{21},a_{22},a_{23},\ldots ,a_{26}\\ a_{31},a_{32},a_{33},\ldots ,a_{36}\\ a_{41},a_{42},a_{43},\ldots ,a_{46}\\ a_{51},a_{52},a_{53},\ldots ,a_{56}\\ a_{61},a_{62},a_{63},\ldots ,a_{66} \end{array}\right],\\ A=\left[\begin{array}{c} 0,1,1,0,0,0\\ 1,0,1,0,0,1\\ 1,1,0,1,1,0\\ 0,0,1,0,1,1\\ 0,0,1,1,0,0\\ 0,1,0,1,0,0 \end{array}\right]. \end{array}$$

*Q*=*AX*, and we only list the first line of *Q*:

(4) $$\begin{array}{c} Q_{1•}=\left[a_{21}+a_{31},a_{22}+a_{32},a_{23}+a_{33},a_{24}+a_{34},a_{25}+a_{35}\right]. \end{array}$$

*Q*_1_ can be expressed as the sum of the feature vectors of the two neighbor nodes of *x*_1_. If the weight matrix is added, the meaning of weighted average can be expressed, which is the convolution operation in the traditional sense. However, there is one point that needs to be explained here. Since the convolution operation is a weighted average of the central node and the surrounding nodes, the above formula does not involve the central node, the adjacency matrix *A* is improved, and the original adjacency matrix *A* is added with its own node.

(5) $$\begin{array}{c} \hat{A}=A+I. \end{array}$$

$\hat{A}$ is normalized to

(6) $$\begin{array}{c} \hat{A}={\hat{D}}^{-1/2}\hat{A}{\hat{D}}^{-1/2},\\ f\left(H^{\left(l\right)},A\right)=\sigma \left({\hat{D}}^{-1/2}\hat{A}{\hat{D}}^{-1/2}H^{\left(l\right)}W^{\left(l\right)}\right). \end{array}$$

**Figure 2:**
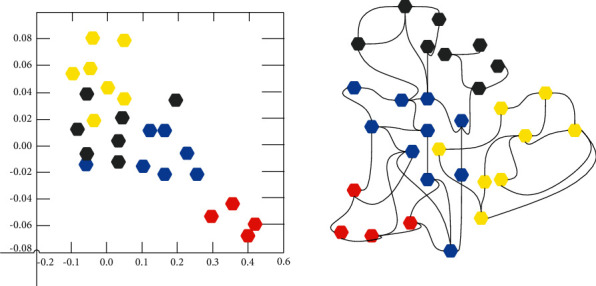
Diagram structure of Taekwondo club.

Although this model is very simple, the model effect is very powerful.  Figure 2 shows the use of a simple GCN model on the famous Taekwondo club data set in this paper. After experimentation, it is found that only 3 levels are needed to classify club members. Moreover, the experimental result of using the identity matrix as the characteristic matrix when the characteristic matrix of the node is not known is also very impressive. For many graph networks, there may be no node characteristics, and graph convolutional neural networks can also be used at this time. The identity matrix I replaces the feature matrix *X* operation.

The second airspace method converts graph structure data into Euclidean space data. First, it selects a representative node sequence from the graph structure and second designs a neighborhood with a fixed convolution kernel size for each node. Then, it converts each graph structure data into Euclidean space data, which can be solved by using traditional convolution operations.

**Figure 3:**
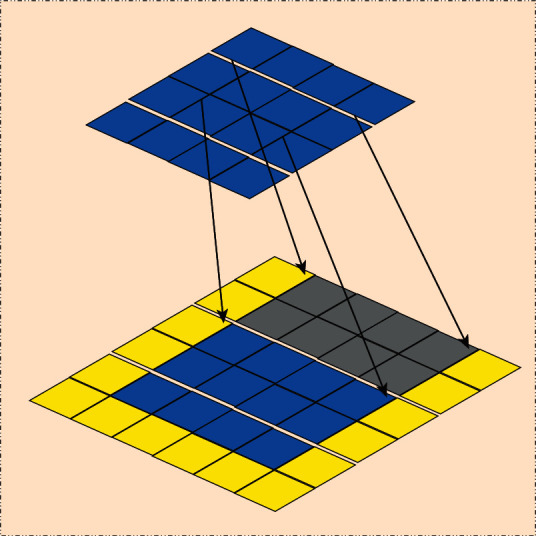
Traditional convolution operation.

**Figure 4:**
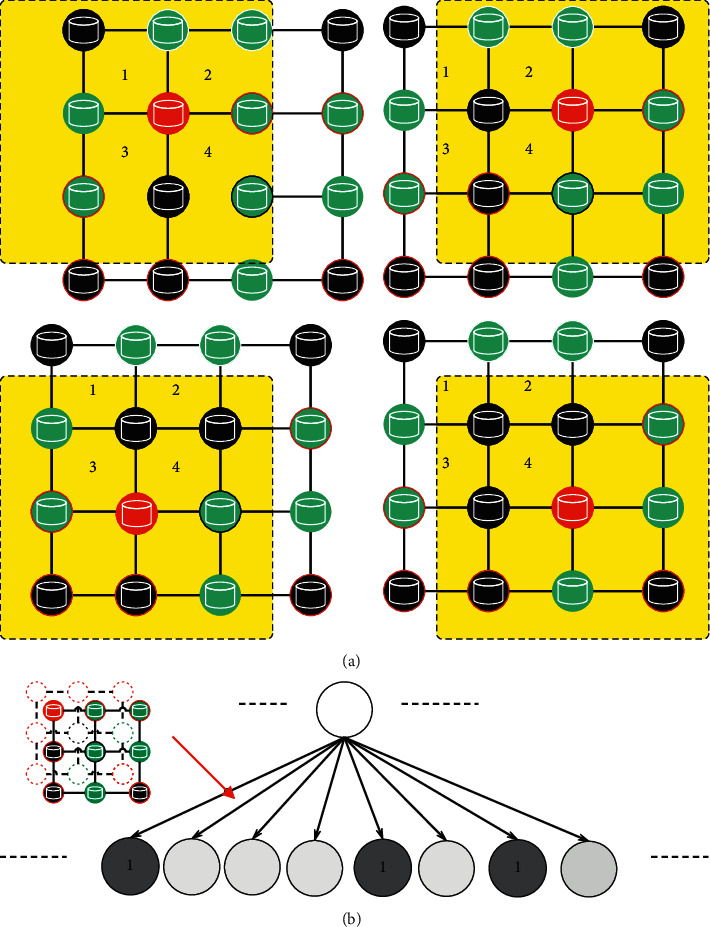
Graph structure transformation.

Each image can be regarded as a special graph, and each pixel in the image can be represented as a node. The convolution operation for each image is the weighted average of *n* central pixels and the surrounding pixels. Therefore, each graph structure can also be regarded as a weighted average of *n* central vertices and corresponding neighborhoods, so that the graph structure can be converted into Euclidean space data. Traditional convolution operation is as shown in Figure 3, and graph structure transformation is as shown in Figure 4.

This paper selects *n* representative nodes, and the node selection is based on the Weisfeiler–Lehman algorithm. It can be understood as calculating the centrality of each node, sorting according to the size of the value, and then taking the first *n* representative nodes.

**Figure 5:**
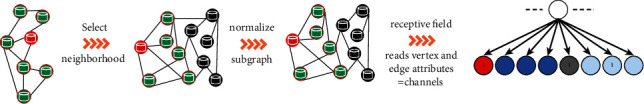
Operation steps.

The neighborhood of the node is constructed by normalizing the neighborhood in the previous step. Normalization makes the nodes of the neighborhood graph have an order so that the unordered graph space is mapped to the vector space in a linear order. Moreover, this paper defines the optimal graph normalization problem and finds the optimal order by optimizing the function. The operation steps are shown in Figure 5.

(7) $$\begin{array}{c} \arg\min_{l}{\text{E}}_{G}\left[\left|d_{A}\left(A^{l}\left(G\right),A^{l}\left(G^{\prime }\right)\right)-d_{G}\left(G,G^{\prime }\right)\right|\right]. \end{array}$$

Each graph structure data can map the disordered neighborhood to the vector space through node selection and node neighborhood construction. Therefore, the method proposed in this paper is to build a bridge between graph structure data and convolutional neural network and solve the transformed Euclidean space data through traditional convolutional neural network.

In the traditional Fourier transform $\hat{f}\left(\omega \right)=\int f\left(t\right)e^{-i\omega t}dt$, the integral operation here can be understood as a linear combination of *f*(*t*) and basis function *e*^−*icx*^. We only need to compare the basis function *h* used here to find the basis function on the graph.

The eigen decomposition of Laplacian matrix *L* is

(8) $$\begin{array}{c} L=U\left[\begin{array}{ccc} \lambda_{1} & \; & \;\\ \; & \ddots & \;\\ \; & \; & \lambda_{n} \end{array}\right]U^{-1}. \end{array}$$

Because *L* is an orthogonal matrix,

(9) $$\begin{array}{c} L=U\left[\begin{array}{ccc} \lambda_{1} & \; & \;\\ \; & \ddots & \;\\ \; & \; & \lambda_{n} \end{array}\right]U^{\text{T}}. \end{array}$$

The characteristic matrix *U* of the Laplacian matrix *L* is the base matrix of the graph convolution, so the graph Fourier transform operation $\hat{f}\left(\omega \right)=\sum_{i=1}f\left(i\right)u_{l}^{*}\left(i\right)$ is converted into a matrix form:

(10) $$\begin{array}{c} f^{*}g=\left[\begin{array}{cccc} u_{1}\left(1\right) & u_{1}\left(2\right) & \cdots & u_{1}\left(N\right)\\ u_{2}\left(1\right) & u_{2}\left(2\right) & \cdots & u_{2}\left(N\right)\\ \vdots & \vdots & \ddots & \vdots \\ u_{N}\left(1\right) & u_{N}\left(2\right) & \cdots & u_{N}\left(N\right) \end{array}\right]\left[\begin{array}{c} f\left(1\right)\\ f\left(2\right)\\ \vdots \\ f\left(N\right) \end{array}\right]. \end{array}$$

Among them, *f* and *g* are the original signals, *F*(*f*) is the Fourier transform of *f*,  *·* represents the product, and ^*∗*^ represents the convolution operation.

(11) $$\begin{array}{c} f^{*}h=F^{-1}\left(\hat{f}\left(w\right)\hat{h}\left(w\right)\right)=\frac{1}{2\pi }\int \hat{f}\left(w\right)\hat{h}\left(w\right)e^{iex}d\omega \;. \end{array}$$

Analogy to $f^{*}h=U\left(\hat{f}\hat{h}\right)$ in the graph, the Fourier transform $\hat{h}\left(\lambda_{l}\right)=\sum_{i=1}^{N}h\left(i\right)u_{l}^{*}\left(i\right)$ of the convolution kernel *h* is written as a diagonal matrix as follows:

(12) $$\begin{array}{c} \hat{h}=\left[\begin{array}{ccc} \hat{h}\left(\lambda_{1}\right) & \; & \;\\ \; & \ddots & \;\\ \; & \; & \hat{h}\left(\lambda_{n}\right) \end{array}\right]. \end{array}$$

Therefore, the generalized convolution on the graph is as follows:

(13) $$\begin{array}{c} f^{*}g=U\left[\begin{array}{ccc} \hat{h}\left(\lambda_{1}\right) & \; & \;\\ \; & \ddots & \;\\ \; & \; & \hat{h}\left(\lambda_{n}\right) \end{array}\right]U^{T}f\text{, }U=\left(\overset{⟶}{u_{1}},\overset{⟶}{u_{2}},\cdots ,\overset{⟶}{u_{n}}\right). \end{array}$$

*U* is the unit eigenvector matrix of the Laplacian matrix. Later, in many spectral domain graph convolution papers, it is often represented by (*f*^*∗*^*h*)_*G*_=*U*((*U*^T^*h*)Θ*U*^*T*^*f*)), where Θ is the Hadamard product.

The setting of the first-generation convolution kernel directly selects the parameter diagonal matrix to represent.

(14) $$\begin{array}{c} g_{\theta }\left(\Lambda \right)=\left[\begin{array}{ccc} \theta_{1} & \; & \;\\ \; & \ddots & \;\\ \; & \; & \theta_{n} \end{array}\right]. \end{array}$$

Among them, Θ(*θ*_1_, *θ*_2_ ⋯ *θ*_*n*_) is *n* arbitrary parameters. It can be seen that the Laplacian matrix needs to be eigen-decomposed when seeking graph convolution.

Due to the large dimension of the Laplacian matrix of the graph data, calculating the eigenvalues will consume a lot of time. Moreover, because the first-generation convolution kernel does not have local features, many scholars have begun to modify the convolution kernel and proposed the second-generation convolution kernel.

(15) $$\begin{array}{c} g_{\theta }\left(\Lambda \right)=\left[\begin{array}{ccc} \overset{\text{K}}{\underset{j=0}{\sum }}\alpha_{j}\lambda_{1}^{j} & \; & \;\\ \; & \ddots & \;\\ \; & \; & \overset{\text{K}}{\underset{j=0}{\sum }}\alpha_{j}\lambda_{n}^{j} \end{array}\right]. \end{array}$$

The ingenious thing is

(16) $$\begin{array}{c} \Lambda^{j}=\left[\begin{array}{ccc} \lambda_{1}^{j} & \; & \;\\ \; & \ddots & \;\\ \; & \; & \lambda_{n}^{j} \end{array}\right]. \end{array}$$

When substituting it into the above formula, we can get

(17) $$\begin{array}{c} g_{\theta }\left(\Lambda \right)=\left[\begin{array}{c} \overset{K}{\underset{j=0}{\sum }}\alpha_{j}\lambda_{1}^{j}\\ \ddots \\ \overset{K}{\underset{j=0}{\sum }}\alpha_{j}\lambda_{n}^{j} \end{array}\right]=\overset{K}{\underset{j=0}{\sum }}\alpha_{j}\Lambda^{j},\\ U\overset{K}{\underset{j=0}{\sum }}\alpha_{j}\Lambda^{j}U^{T}=\overset{K}{\underset{j=0}{\sum }}\alpha_{j}U\Lambda^{j}U^{T}=\overset{K}{\underset{j=0}{\sum }}\alpha_{j}L^{j}. \end{array}$$

Therefore, the convolution operation is transformed into

(18) $$\begin{array}{c} y_{\text{output }}=\sigma \left(\overset{K}{\underset{j=0}{\sum }}\alpha_{j}L^{j}x\right). \end{array}$$

Compared with the first-generation convolution kernel, the second-generation convolution kernel reduces the number of parameters, reduces the complexity of the parameters, and speeds up the calculation. The convolution operation avoids the eigen decomposition of the Laplacian matrix, and the convolution kernel adds the local features of each node.

In addition to cleverly designing the convolution kernel to avoid the eigendecomposition of the Laplacian matrix, Chebyshev polynomials can also be used to fit the convolution kernel, which is a widely used method in GCN papers. The third-generation graph convolutional network (GCN) is proposed.

The first-generation convolution kernel is

(19) $$\begin{array}{c} y_{\text{output }}=\sigma \left(Ug_{\theta }\left(\Lambda \right)U^{\text{T}}x\right). \end{array}$$

Among them, *U* is the matrix formed by the eigenvectors of the Laplacian matrix.

Using the Chebyshev polynomial instead of the original convolution kernel, we can get $g_{\theta }\left(\Lambda \right)=\sum_{k=0}^{K-1}\beta_{k}{\text{T}}_{k}\left(\tilde{\Lambda }\right)\;$, where *T*_*k*_(·) is the k-order Chebyshev polynomial, *β*_*k*_ is the corresponding parameter, and Λ is the diagonal matrix of eigenvalues.

(20) $$\begin{array}{c} \tilde{\Lambda }=\frac{2\Lambda }{\lambda_{\max}}-I. \end{array}$$

It can be seen that the elements of the characteristic diagonal matrix are limited to [0, 1] at this time. The reason for this operation is that the Chebyshev polynomial has a domain of [−1, 1].

(21) $$\begin{array}{c} y_{\text{output }}=\sigma \left(U\overset{K-1}{\underset{k=0}{\sum }}\beta_{k}{\text{T}}_{k}\left(\tilde{\Lambda }\right)U^{\text{T}}x\;\right),\\ y_{\text{owput }}=\sigma \left(\overset{K-1}{\underset{k=0}{\sum }}\beta_{k}{\text{T}}_{k}\left(\tilde{U\Lambda }U^{\text{T}}\right)x|\;\right),\\ y_{\text{oupput }}=\sigma \left(\overset{K-1}{\underset{k=0}{\sum }}\beta_{k}{\text{T}}_{k}\left(\tilde{L}\right)x|\;\right). \end{array}$$

Among them,

(22) $$\begin{array}{c} \tilde{L}=\frac{2L}{\lambda_{\max}}-I,T_{k}\left(\tilde{L}\right)=2\;\tilde{L}T_{K-1}\left(\tilde{L}\right)-T_{k-2}\left(\tilde{L}\right),T_{0}\left(\tilde{L}\right)=I,T_{1}\left(\tilde{L}\right)=\tilde{L},L=I-D^{-\frac{1}{2}}AD^{-\frac{1}{2}}. \end{array}$$

We can see that

1. Chebyshev polynomial as a convolution kernel avoids the eigen decomposition of the Laplacian matrix.
2. By introducing the adjacency relationship of the graph, the convolution kernel has local features. Therefore, the graph convolution model based on the spectral domain method is a further promotion of the basis function of the Fourier transform and the convolution kernel of the convolution operation.

The data in the social recommendation system can be naturally expressed as a user-user social graph and a user-item graph, and the BP neural network can integrate the node information in the above two graph structures. Therefore, BP neural network is widely used in product recommendation, movie recommendation, and other fields. User Modeling integrates the user information extracted from the user's social network (which can be understood as the characteristics of friends in the user's social relationship and reflects some of the characteristics of the user) with the user information extracted from the user-item graph structure (can be understood as the user's rating of the item and reflects the user's characteristics).

**Figure 6:**
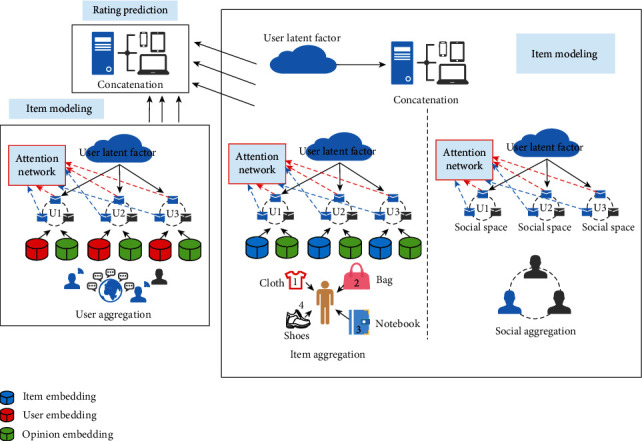
Graphrec model.

The Graphrec model includes User Modeling, Item Modeling, and Rating Prediction (Figure 6). Item Modeling integrates the characteristics of the item with the characteristics of the users participating in the evaluation of the item to reflect the characteristics of the item. Rating Prediction combines the user potential features extracted from the User Modeling and Item Modeling described above with the potential features of the item to predict the rating of each item.

The following is a detailed introduction to the content of the paper.

### 3.1. User Modeling

In order to understand the potential characteristics of users, the user model is divided into two parts, user-item graph and social network, and the two parts are described separately.

#### 3.1.1. User-Item Graph Model

(23) $$\begin{array}{c} h_{i}^{I}=\sigma \left(W\cdot {\text{ aggre }}_{\text{item }}\left(\left\{x_{ia},\forall a\in C\left(i\right)\right\}\right)+b\right). \end{array}$$

Among them, *C*(*i*) is the item evaluated by user *u*(*i*), *W*, *b* is the weight and bias of the neural network, and *σ* is a nonlinear function.

Users will express their opinions when scoring items, so items and item scoring can help users model.

(24) $$\begin{array}{c} x_{ia}=g_{v}\left(\left[q_{a}⊕e_{r}\right]\right). \end{array}$$

Among them, *x*_*ia*_ is the integration of users and items, *x*_*ia*_ is the interactive representation of each item and the score, *q*_*a*_ is the feature vector of the item, *e*_*r*_ is the feature of the five evaluation levels, and ⊕ is the connection of two vectors.

Generally, the abovementioned aggretions fusion function directly integrates multiple items evaluated by each user on an average basis, which as follows:

(25) $$\begin{array}{c} h_{i}^{l}=\sigma \left(W\cdot \left\{\underset{acc\left(t\right)}{\sum }\alpha_{i}x_{ia}\right\}+b\right),\\ \alpha_{i}=\frac{1}{\left|C\left(i\right)\right|}. \end{array}$$

Because the proportion of each item is different for users, it is not possible to directly calculate the average integration. In order to make up for the lack of average integration, the attention mechanism is introduced.

(26) $$\begin{array}{c} h_{i}^{l}=\sigma \left(W\cdot \left\{\underset{acC\left(i\right)}{\sum }\alpha_{ia}x_{ia}\right\}+b\right), \end{array}$$

where *α*_*ia*_ is the weight of the attention mechanism.

(27) $$\begin{array}{c} \alpha_{ia}^{*}=\left(w_{2}^{T}\cdot \sigma \left(W_{1}\cdot \left[x_{ia}⊕p_{i}\right]\right)+b_{1}\right)+b_{2},\\ \alpha_{ia}=\frac{\exp\left(\alpha_{ia}^{*}\right)}{\sum_{a\epsilon C\left(i\right)}\exp\left(\alpha_{ia}^{*}\right)}. \end{array}$$

Among them, *p*^*i*^ is the feature vector of user *u*_*i*_, the interaction of items and ratings is connected with the feature vector of the user, and *W*_1_ and *w*_2_^*T*^, *b*_1_,  and *b*_2_ are weights and biases. The Softmax function is used to normalize the above attention weights to obtain the final attention weights, which can be understood as the contribution of the interaction to the user-item space user latent factor.

In the interaction between users and friends, it can be found that users' evaluations of products are similar to their own friends' evaluations. Therefore, this paper proposes to obtain the potential characteristics of the user by fusing the evaluation of the user's friends on the project and the characteristics of the project itself and design the following fusion function:

(28) $$\begin{array}{c} h_{i}^{S}=\sigma \left(W\cdot {\text{ Aggre }}_{\text{neighhors }}\left(\left\{h_{o}^{I},\;\forall o\in N\left(i\right)\right\}\right)+b\right),\\ h_{i}^{S}=\sigma \left(W\cdot \left\{\underset{\text{oeN}\left(i\right)}{\sum }\beta_{io}h_{o}^{I}\right\}+b\right). \end{array}$$

Since different friends have different influences on users, this paper introduces an attention mechanism to users' friends.

(29) $$\begin{array}{c} \beta_{io}^{*}=w_{2}^{T}\cdot \sigma \left(W_{1}\cdot \left[h_{o}^{I}⊕p_{i}\right]+b_{1}\right)+b_{2},\\ \beta_{io}=\frac{\exp\left(\beta_{io}^{*}\right)}{\sum_{o\in N\left(i\right)}\exp\left(\beta_{io}^{*}\right)}. \end{array}$$

The potential user characteristics obtained by the user-item graph model and the user potential characteristics obtained by the user social network model are merged, and the two parts of user characteristics are extracted through the full convolutional neural network to obtain the final user potential characteristics.

(30) $$\begin{array}{c} c_{1}=\left[h_{i}^{l}⊕h_{i}^{s}\right],\\ c_{2}=\sigma \left(W_{2}\cdot c_{1}+b_{2}\right),\\ \cdots \\ h_{i}=\sigma \left(W_{l}\cdot c_{l-1}+b_{l}\right). \end{array}$$

### 3.2. Item Modeling

The project model is to extract the potential features of the project and extract the potential features of the project by fusing the characteristics of the users participating in the project evaluation and the user's score.

(31) $$\begin{array}{c} f_{jt}=g_{ut}\left(\left[p_{t}⊕e_{r}\right]\right). \end{array}$$

Each user feature is fused, and the fusion function is designed as follows:

(32) $$\begin{array}{c} z_{j}=\sigma \left(W\cdot {\text{ Aggre }}_{\text{users }}\left(\left\{f_{jt},\;\forall t\in B\left(j\right)\right\}+b\right)\right.,\\ z_{j}=\sigma \left(W\cdot \left\{\underset{t\in B\left(j\right)}{\sum }\mu_{jt}f_{jt}\right\}+b\right). \end{array}$$

Since each user has a different proportion of item feature extraction, this paper adds an attention mechanism.

(33) $$\begin{array}{c} \mu_{jt}^{*}=w_{2}^{T}\cdot \sigma \left(W_{1}\cdot \left[f_{jt}⊕q_{j}\right]+b_{1}\right)+b_{2},\\ \mu_{jt}=\frac{\exp\left(\mu_{jt}^{*}\right)}{\sum_{t\in B\left(j\right)}\exp\left(\beta_{jt}^{*}\right)}. \end{array}$$

### 3.3. Rating Prediction

Recommendation can be simplified as a matching problem between users and items, which matches suitable items for suitable users and derives users' predictions for items. Proved that the full convolutional layer (MLP) can approximate any measurable function with arbitrary accuracy, so similarity can also be learned using the full convolutional layer (MLP).

(34) $$\begin{array}{c} g_{1}=\left[h_{i}⊕z_{j}\right],\\ g_{2}=\sigma \left(W_{2}\cdot g_{1}+b_{2}\right),\\ \cdots \\ g_{l-1}=\sigma \left(W_{l}\cdot g_{l-1}+b_{l}\right),\\ r_{ij}^{\prime }=w^{T}\cdot g_{l-1}. \end{array}$$

### 3.4. Loss Function

(35) $$\begin{array}{c} L\text{ oss }=\frac{1}{2\left|O\right|}\underset{i,j\in O}{\sum }{\left(r_{ij}^{\prime }-r_{ij}\right)}^{2}. \end{array}$$

Among them, *O* is the set of tuples (*i*, *j*), where the tuples represent the user *u*_*i*_ and the rated item *v*_*j*_, so this loss function calculates the loss for all rated items and does not consider the loss for the unrated items.

## 4. Selection Model of Cultural and Creative Tourist Attractions Based on BP Network

**Figure 7:**
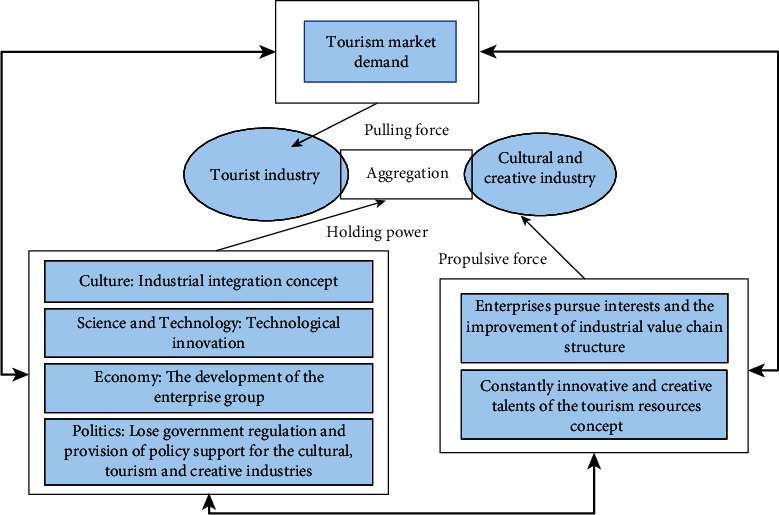
Integration mechanism of tourism industry and cultural creative industry.

From the above introduction to system theory, we can find that the essence of a system is a process. This process has always been in dynamic change and development, and the system structure is the manifestation of this dynamic change. Under the guidance of this theory, this paper combines the economic “supply-demand” structure theory to construct a dynamic process of the integration of cultural and creative industries and tourism industries. Based on the perspective of system theory, the concept of industry is defined as a systematic combination of related elements such as technology, products, enterprises, markets, and systems. Different industrial factors interact with each other, continuously differentiate and restructure, and promote the development of the industrial system. It can be said that the integration mechanism of industries is the result of the evolution of different divisions of labor between industries or within industries. This evolution process is shown in Figure 7.

The interrelationship and mutual promotion of cultural and creative industries and tourism industries constitute a complex and changeable dynamic system. The main driving force of this dynamic system comes from the continuous expansion of the demand for tourism creativity and the continuous increase of the supply of tourism creativity products. This interactive dynamic system can better promote industrial integration with the support of regional cultural creativity and mature tourism market. The driving force generated by the interaction between the demand thrust and the supply pull and the interaction of various factors in the external market environment constitutes the main driving force for the industrial integration of the cultural and creative industries and the tourism industry.

**Figure 8:**
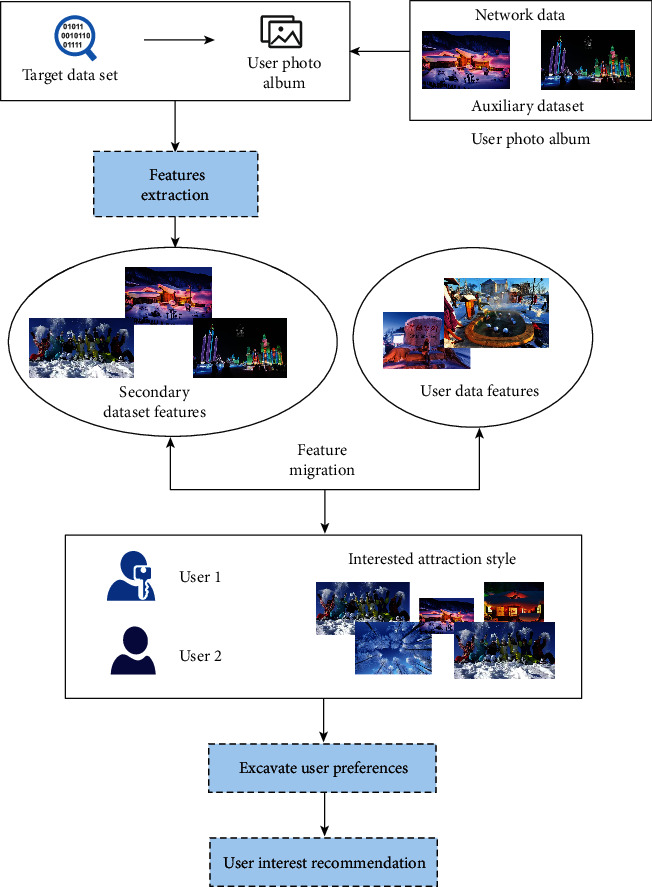
Block diagram of the personalized recommendation model for cultural and creative tourist attractions.

The recommendation performance of the cultural and creative personalized tourist attraction recommendation system will be greatly reduced, and manual labeling of training data is time-consuming and laborious. Therefore, it is considered to introduce a labeled auxiliary data set related to the target data set to solve the problem of the lack of labeled data in the target data. However, there is a distribution difference between the auxiliary data set and the target data set, so the data distribution difference between the two must be eliminated before it can be used to train a personalized travel recommendation system. Therefore, a personalized recommendation model for tourist attractions based on domain adaptation is proposed. Domain adaptation can effectively reduce the distribution difference between different data. The block diagram of the personalized recommendation model for cultural and creative tourist attractions based on domain adaptation is shown in Figure 8.

**Figure 9:**
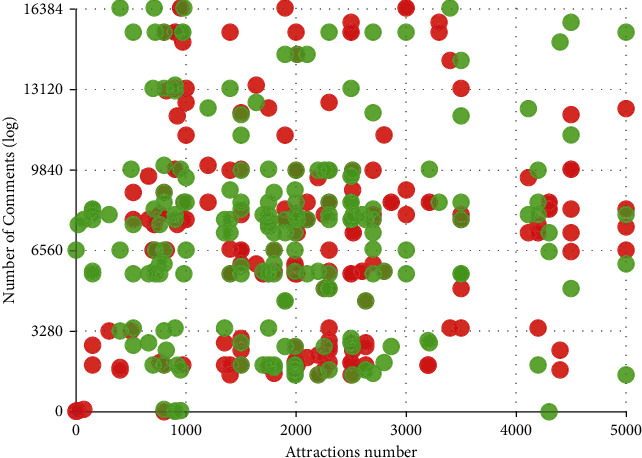
Reference diagram for setting the threshold of the dividing line of tourist attractions.

Figure 9 is a schematic diagram of setting the time boundary threshold *t* and the popular/not popular boundary threshold *b*. It can be seen that the comment values of a large number of scenic spots are concentrated in the lower area of the figure. This distribution of the number of reviews is consistent with the “long-tail effect” in the distribution of scenic spots mentioned in Section 4 and is consistent with the setting of using the number of reviews to reflect the popularity of scenic spots. It is reasonable to set the *b* value in the interval shown in the figure. In order to avoid the excessive subjective influence caused by the need to manually set the *b* value, this paper considers setting the experiment to select multiple thresholds *b* and analyzes the influence of the scenic spot popularity prediction under different *b* values.

**Figure 10:**
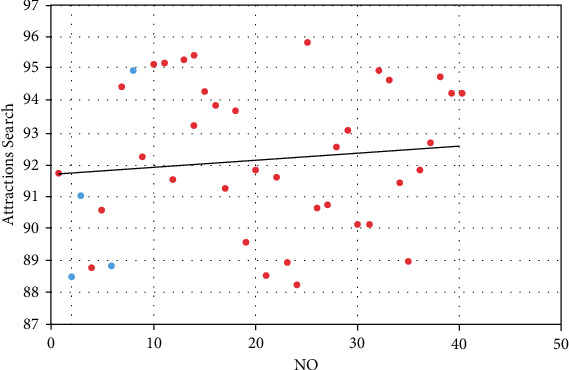
Statistics diagram of search results of the cultural and creative tourist attractions selection model based on BP network.

Based on the above analysis, the effect of this model is verified. The system model in this paper is mainly applied to the selection and search of cultural and creative tourist attractions. Therefore, this paper conducts a simulation experiment on the search effect of the cultural and creative tourist attractions selection model based on the BP network and obtains the results shown in Table 1 and Figure 10.

**Figure 11:**
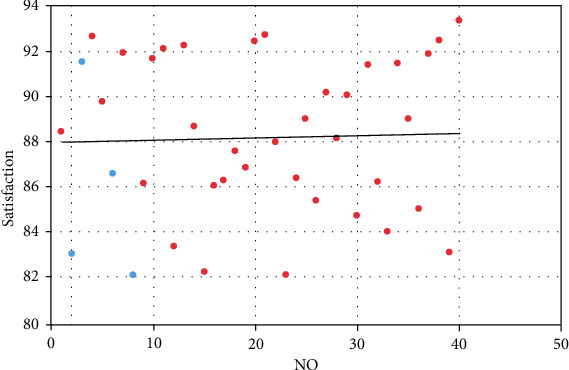
Statistical diagram of user satisfaction of the model.

The above research verifies the search effect of the cultural and creative tourist attractions selection system based on BP network. On this basis, a user satisfaction survey is conducted on the model in this paper, and the results are shown in Table 2 and Figure 11.

From the above and the research results, we can see that the cultural and creative tourist attractions selection search system based on BP network has good user satisfaction. On the whole, the cultural and creative tourist attraction selection search system proposed in this paper based on the BP network has a good selection effect of tourist attractions and has an important role in promoting the development of cultural and creative tourism.

## 5. Conclusion

The development of China's cultural industry has entered a “new stage,” and the innovative design of regional tourism cultural and creative products in the cultural industry has risen from corporate behavior to an important position in the national economy. For this reason, the focus of designers has shifted from traditional products to innovative products that highlight the five thousand years of Chinese culture and characteristic regional culture, which makes the cultural and creative industry hot. Chinese history and culture have a long history and a history of 5,000 years. The harmonious development of society and nature is based on the basic condition that the diversity of culture and nature is equally important. This paper applies the BP network to the selection and exploration of cultural and creative tourist attractions and builds an intelligent system based on the actual situation. Through experimental research, it can be seen that the cultural and creative tourist attraction selection search system proposed in this paper based on the BP network has a good selection effect of tourist attractions and has an important role in promoting the development of cultural and creative tourism.

## Figures and Tables

**Table 1:** Search results of the cultural and creative tourist attractions selection model based on BP network.

No.	Attractions search	No.	Attractions search
1	91.70	21	88.49
2	88.46	22	91.58
3	91.00	23	88.91
4	88.75	24	88.20
5	90.53	25	95.82
6	88.78	26	90.62
7	94.41	27	90.74
8	94.92	28	92.49
9	92.25	29	93.05
10	95.13	30	90.12
11	95.17	31	90.12
12	91.50	32	94.91
13	95.27	33	94.66
14	95.43	34	91.40
15	94.27	35	88.94
16	93.82	36	91.83
17	91.22	37	92.65
18	93.70	38	94.74
19	89.54	39	94.22
20	91.77	40	94.23

**Table 2:** User satisfaction of the model.

No.	Satisfaction	No.	Satisfaction
1	88.41	21	92.64
2	83.13	22	87.99
3	91.48	23	82.11
4	92.59	24	86.38
5	89.73	25	88.98
6	86.58	26	85.43
7	91.90	27	90.18
8	82.18	28	88.10
9	86.12	29	90.03
10	91.63	30	84.70
11	92.07	31	91.36
12	83.39	32	86.21
13	92.24	33	84.02
14	88.62	34	91.44
15	82.25	35	88.98
16	86.05	36	85.03
17	86.27	37	91.84
18	87.57	38	92.45
19	86.85	39	83.13
20	92.41	40	93.31

## Data Availability

The labeled dataset used to support the findings of this study is available from the corresponding author upon request.
